# I-Impute: a self-consistent method to impute single cell RNA sequencing data

**DOI:** 10.1186/s12864-020-07007-w

**Published:** 2020-11-18

**Authors:** Xikang Feng, Lingxi Chen, Zishuai Wang, Shuai Cheng Li

**Affiliations:** 1 School of Software, Northwestern Polytechnical University, Xi’an, Shaanxi, 710072 China; 2 Department of Computer Science, City University of Hong Kong, Tat Chee Avenue, Kowloon, Hong Kong, China; 3 Department of Biomedical Engineering, City University of Hong Kong, Tat Chee Avenue, Kowloon, Hong Kong, China

**Keywords:** scRNA-seq, Imputation, Self-consistency, Cell subpopulation identification

## Abstract

**Background:**

Single-cell RNA-sequencing (scRNA-seq) is becoming indispensable in the study of cell-specific transcriptomes. However, in scRNA-seq techniques, only a small fraction of the genes are captured due to “dropout” events. These dropout events require intensive treatment when analyzing scRNA-seq data. For example, imputation tools have been proposed to estimate dropout events and de-noise data. The performance of these imputation tools are often evaluated, or fine-tuned, using various clustering criteria based on ground-truth cell subgroup labels. This limits their effectiveness in the cases where we lack cell subgroup knowledge. We consider an alternative strategy which requires the imputation to follow a “self-consistency” principle; that is, the imputation process is to refine its results until there is no internal inconsistency or dropouts from the data.

**Results:**

We propose the use of “self-consistency” as a main criteria in performing imputation. To demonstrate this principle we devised I-Impute, a “self-consistent” method, to impute scRNA-seq data. I-Impute optimizes continuous similarities and dropout probabilities, in iterative refinements until a self-consistent imputation is reached. On the in silico data sets, I-Impute exhibited the highest Pearson correlations for different dropout rates consistently compared with the state-of-art methods SAVER and scImpute. Furthermore, we collected three wetlab datasets, mouse bladder cells dataset, embryonic stem cells dataset, and aortic leukocyte cells dataset, to evaluate the tools. I-Impute exhibited feasible cell subpopulation discovery efficacy on all the three datasets. It achieves the highest clustering accuracy compared with SAVER and scImpute.

**Conclusions:**

A strategy based on “self-consistency”, captured through our method, I-Impute, gave imputation results better than the state-of-the-art tools. Source code of I-Impute can be accessed at https://github.com/xikanfeng2/I-Impute.

## Background

Single-cell RNA-sequencing (scRNA-seq) is becoming indispensable in studying the landscapes of cell-specific transcriptomes [1]. It demonstrates robust efficacy in capturing transcriptome-wide cell-to-cell heterogeneity with high resolution [2–5]. With meta information such as time series or patient histology, scRNA-seq has the potential to decipher the underlying patterns in cell cycles [6–8], complex diseases [9–11], and cancers [8, 12–16].

As with other sequencing techniques, scRNA-seq produces a count matrix which captures expression profiles, with genes as rows, cells as columns, and the gene counts as the matrix elements. scRNA-seq only captures a small fraction of the genes due to “dropout” events. That is, it produces a zero-inflated count matrix where only about 10% entries are non-zero values [17]. This is mainly due to the missing of truly expressed transcripts from some cells during sequencing. The dropout rate is protocol-dependent [18]. When analyzing scRNA-seq data, the excess zero counts from dropout events needs to be remedied. Otherwise, the zero count distribution from different protocols may lead to diverging potency, which will affect downstream analyses [18], such as clustering, cell type recognition, dimension reduction, differential gene expression analysis, identification of cell specific genes and reconstruction of differentiation trajectory on zero-inflated single-cell gene expression data [18]. The correctness of all these analyses are contingent on the correctness of the expression profile.

As a remedy, downstream scRNA-seq-based analyses such as clustering, cell type recognition, and dimension reduction, can be adapted to implicitly incorporate considerations for dropout events [19–22]. On the other hand, dropout events can be treated prior to downstream analysis with scRNA-seq imputation tools. Two such leading tools are SAVER and scImpute. SAVER [23] imputes by borrowing information across genes using a Bayesian approach which estimates the expression levels. It aims to reduce meaningless biological variation and retain valuable biological variation. One caveat is that SAVER would unfairly adjust all gene expression levels including the actual non-expression of genes, hence possibly interject new biases and abolish real biological meanings. scImpute [18] is designed to first identify dropout values with Gamma-Normal mixture model, and then impute the dropout events by borrowing information from similar cells, with the expression level of un-dropout events unchanged. It automatically excludes the outlier cells and their gene information, which are likely to influence the original imputation values. While scImpute is able to avoid the problem which SAVER faces, it is not good with extremely sparse datasets.

On in silico data where the ground truth counts are known, the root mean square error (RMSE) between imputed and ground truth entries is the most common metrics for imputation evaluation [24]. For wetlab data sets, the ground truth counts for missing events are unknown. One common practice is to randomly remove non-zeros entries and employ an imputation tool to impute these removed entries. Then, the RMSE for the removed entries is calculated as a criterion to evaluate the performance of the imputation [24, 25]. Another common practice is to implicitly validate imputation efficacy by checking whether the imputation improves the downstream analysis result. This check, on the other hand, typically requires additional knowledge. For instance, clustering measurements such as adjusted Rand index (ARI), normalized mutual information (NMI), silhouette width (SW), and within-cluster sum of squares are commonly adopted for scRNA-seq imputation evaluation [18, 26], but these evaluations all require the true cluster labels, which are often hard to obtain.

As an explicit measurement, *imputation consistency* has been discussed in several studies. Buuren et al. [27] stated that the imputed entries should remain internal homogeneous to the non-missing data. Liang et al. adopts consistent estimate after imputation step for high-dimensional data [28]. Here, we propose a new interpretation for imputation consistency. As a reliable imputation tool should assume its output contains no dropout or errors. We want the imputation tool to be consistent in its output: If we are to feed the output to the imputation tool again after eliminating a number of entries, the tool should be able to reproduce these entries. We refer this property as *self-consistency*.

Therefore, in this study, to study the effects of the new criterion, we developed a self-consistent method called I-Impute for scRNA-seq data imputation. We compared I-Impute with the state-of-the-art imputation tools, by evaluating their imputation performance as well as their self-consistency. On the in silico data sets, I-Impute exhibited consistently the highest Pearson correlations for different dropout rates compared to SAVER and scImpute. Furthermore, several discrete cell subpopulations have been reported in scRNA-Seq data collected from the wet lab; the identification of subpopulations of cells is crucial [29]. Here, we collected three wetlab datasets, mouse bladder cells dataset, embryonic stem (ES) cells dataset, and aortic leukocyte cells dataset to evaluate the tools. I-Impute exhibited feasible cell subpopulation discovery efficacy on all the three datasets. It achieves the highest clustering accuracy compared to SAVER and scImpute.

## Results

### Evaluating the self-consistency of existing imputation tools in synthetic data

To evaluate the imputation tools, we applied the R package Splatter [30] to generate scRNA-seq reads count data. We simulated 150 cells of three groups, each with 2,000 genes. Then we generated three sparse matrices by setting the dropout rates as 88.45%, 63.29%, and 45.16%; and their corresponding zero rates are 90.87%, 70.98%, and 56.65%, respectively.

We first validated whether the existing imputation tools are self-consistent. We consider the imputation process as a complex function *f*:*x*→*x* that maps the zero-inflated matrix into an output matrix of the same shape. We say that *f* is *self-consistent* if and only if the root mean square error (RMSE) between *x* and *f*(*x*) is less than a pre-determined threshold *θ*, that is, ||*x*−*f*(*x*)||_2_≤*θ*. The results are shown in Table 1. We found that SAVER and scImpute are not self-consistent. scImpute has RMSE values of 7.346 at 88.45% dropout data, 0.2392 at 63.29% dropout data, and 0.2677 at 45.16% dropout data. For these data sets, SAVER has RMSE value of 0.5613, 1.0245, and 1.3561 respectively. Nevertheless, when ground truth group labels are incorporated, traditional evaluation metrics show SAVER to outperformed scImpute with respect to adjusted Rand index (ARI), normalized mutual information (NMI), and silhouette width (SW) (see Additional file 1, Table S1).

**Table 1:** Self-consistency on synthetic data. NA denotes not applicable. *θ*=0.1

	SAVER	scImpute	I-Impute
88.45% dropout	0.5613	7.3460	0.0936
Self-consistent (<*θ*)	x	x	✓
63.29% dropout	1.0245	0.2392	0.0806
Self-consistent (<*θ*)	x	x	✓
45.16% dropout	1.3561	0.2677	0.0381
Self-consistent (<*θ*)	x	x	✓

**Fig. 1:**
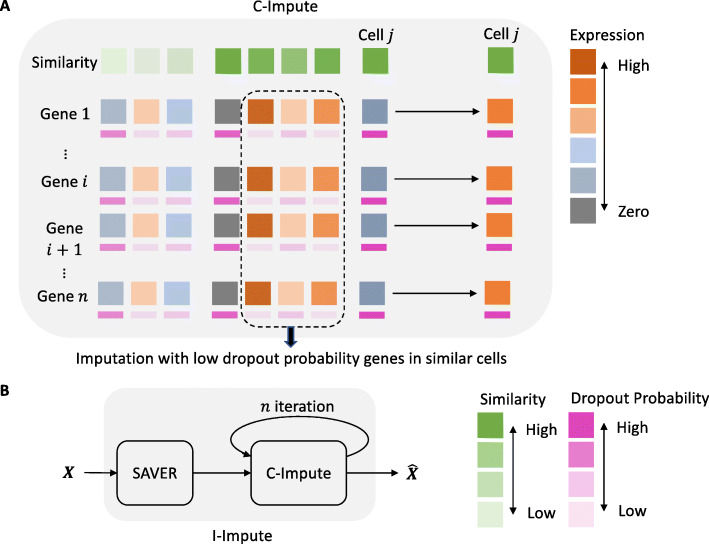
Illustration of I-Impute architecture

Our tool, I-Impute, is constructed on both the principle of self-consistency as well as to optimizing the existing imputation metrics (ARI, NMI, and SW). As illustrated in Fig. 1a, I-Impute first calls an internal subroutine (called C-Impute), which uses continuous similarities and dropout probabilities to infer missing entries. Then, I-Impute invokes SAVER as a subroutine to preprocess the data. Finally, it deploys C-Impute iteratively on the processed data (see Fig. 1b). As illustrated in Additional file 1, Fig. S1, after some number of iterations, the RMSE of I-Impute approaches to below 0.1, which is much smaller than SAVER and scImpute. That is, assume *θ*=0.1, the imputed result converges to a self-consistent matrix, with RMSE values of 0.0936, 0.0806, and 0.0381 in three synthetic datasets, respectively (see Table 1).

### I-Impute recovers gene expression affected by dropouts in synthetic data

**Fig. 2:**
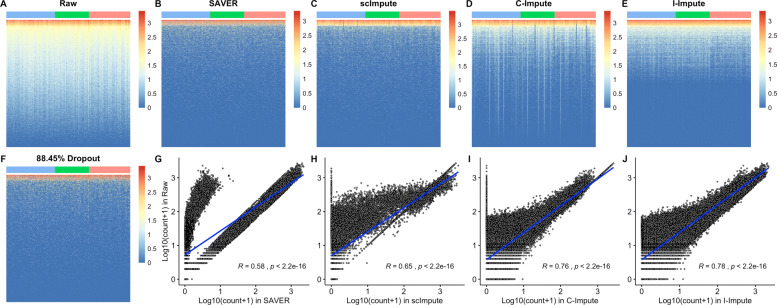
Imputation performance on synthetic data. **a**-**f** Heatmap plots. Blue, green, and red tiles represent different cell groups **g**-**j** Scatter plots, Pearson correlation between ground-truth entries and imputed values are calculated

To validate the performance of I-Impute, we plotted the heatmap of the raw matrix, the 88.45% dropout matrix, and the imputed matrices, respectively (see Fig. 2a-f). I-Impute’s output are closest to the raw matrices, compared to SAVER, scImpute, and C-Impute. As illustrated in Fig. 2g, SAVER failed in reproducing many entries in the raw matrices, leading to the lowest Pearson correlation 0.58 between its output and the ground truth. scImpute and C-Impute changed some highly expressed elements into zero, hence introducing new bias after imputation (see Fig. 2h-i). With no extreme pull-down or pull-up prediction, I-Impute exhibited the most robust recovery power, with the highest Pearson correlation 0.78 (see Fig. 2j). On data with 63.29% and 45.16% dropout rate, I-Impute also gave the highest Pearson correlation of 0.90 and 0.94, respectively (see Additional file 1, Table S4).

**Fig. 3:**
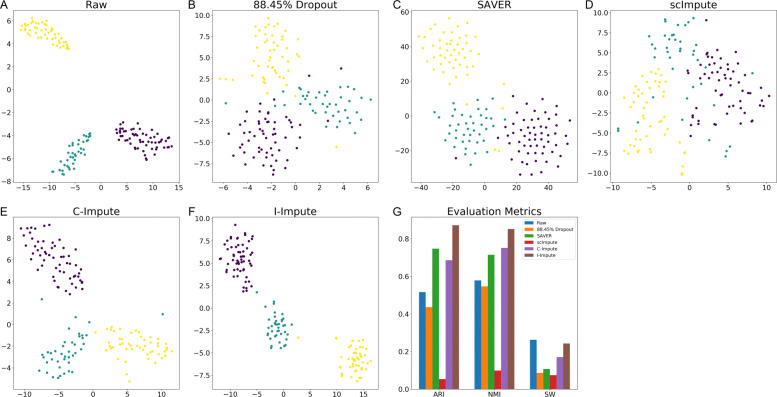
Imputation performance on synthetic data. **a**-**f** t-SNE plots **g** evaluation metrics

The t-SNE embedding plots of the raw matrix, 88.45% dropout matrix, and recovered matrices show that SAVER, C-Impute, and I-Impute recover the missing entries, while preserving cell subgroups structures well (see Fig. 3a-f). Silhouette width (SW) further validated that the in-group similarity and out-group separation were enhanced after the imputation by SAVER, C-Impute, and I-Impute. That is, the average silhouette value increased from 0.0862 (dropout data) to 0.1075 (SAVER), 0.1705 (C-Impute), and 0.2429 (I-Impute), respectively (see Additional file 1, Table S1). Figure 3g demonstrates that I-Impute achieves the most noticeable improvement, while scImpute illustrates lower SW values than dropout data. Next, we applied hierarchical clustering into all matrices, and computed the adjusted Rand index (ARI) and normalized mutual information (NMI) to evaluate the clustering accuracy. ARI and NMI measure the overlap between the inferred groups and ground-truth clusters; a score of 0 implies random labeling while 1 indicates perfect inference. In Fig. 3g, I-Impute outperforms all other tools and exhibits the best sub-population identification strength, with the highest clustering accuracy (ARI: 0.8721, NMI: 0.8521, see Additional file 1, Table S1). Experiments on data sets of 63.2% and 45.16% dropout rate also proved that I-Impute produced the best recovered matrices; with ARI 1.0, NMI 1.0, SW 0.3908 for 63.2% dropout rate, and ARI 0.9801, NMI 0.9710, and SW 0.4123 for 45.16% dropout rate (see Additional file 1, Table S2-S3).

Overall, the synthetic experiment demonstrates that by incorporating C-Impute to refine the SAVER processed data iteratively, I-Impute is able to mitigate the inconsistency in SAVER’s result and this resulted in improved imputation.

### I-Impute promotes cell subpopulation identification in real data sets

**Fig. 4:**
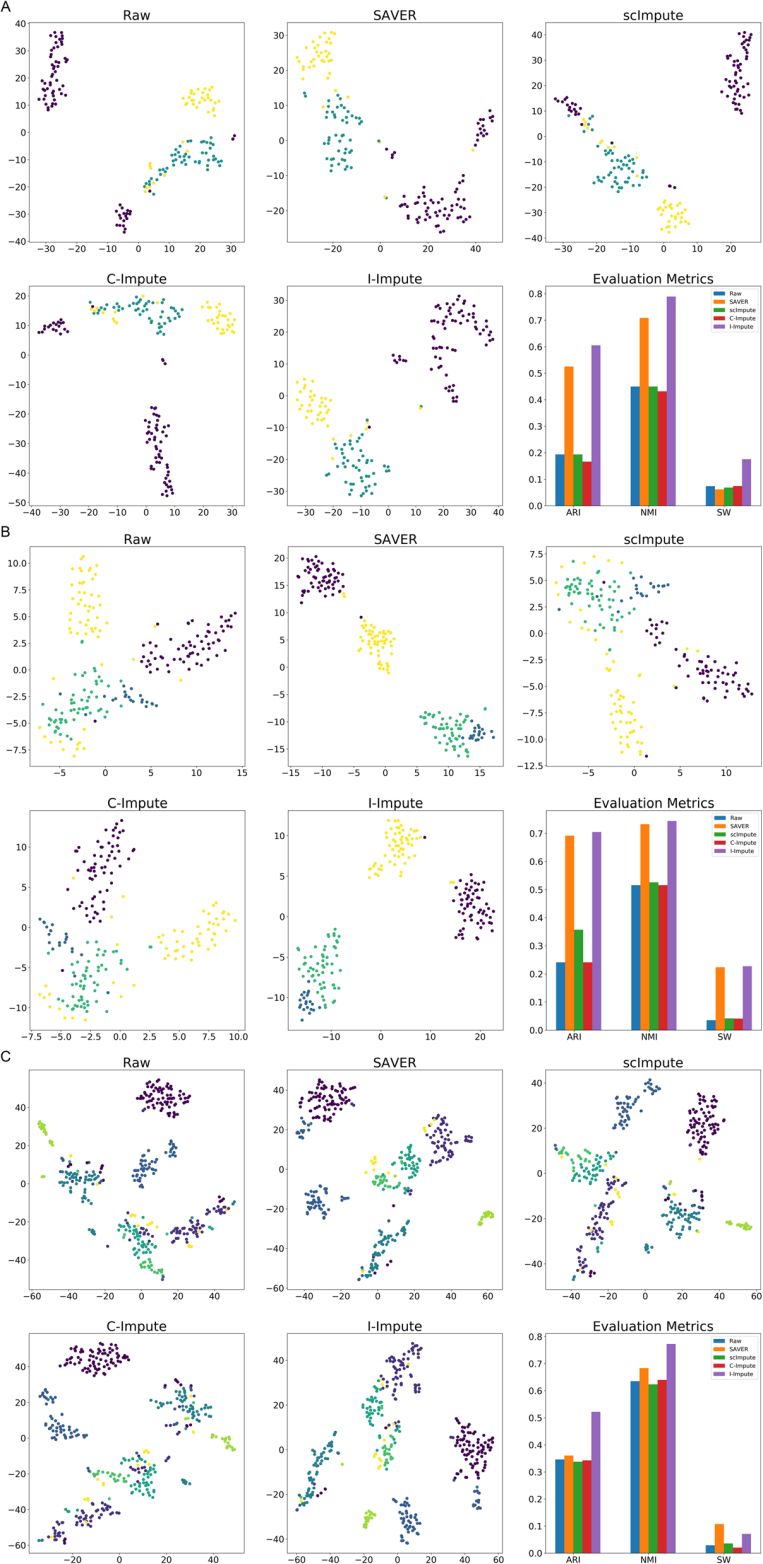
Imputation performance on real datasets. **a**-**c** t-SNE plots and evaluation metrics for mouse bladder cells, embryonicstem cells, and aortic leukocyte cells, respectively

To examine the effects of I-Impute on the identification of cell sub-populations, we performed tests on three real scRNA-Seq datasets. The first test involves a dataset of mouse Bladder cells which contains 162 cells of three cell types. Due to dropout events, 73.5% of the read counts in the raw count matrix are zeros. We evaluated the imputation power by reviewing the tSNE embedding result and silhouette width (SW). ScImpute mixes part of Unknown-type cells (purple dots) with the Fibroblasts-1 cells (blue dots) and Fibroblasts-2 cells (yellow dots); SAVER, C-Impute, and I-Impute distinguish the Unknown-type cells from Fibroblasts-1 cells and Fibroblasts-2 cells well. Compared with raw and other imputed data, I-Impute produced the most compact clusters with highest silhouette width of 0.1758 (Fig. 4a). We then compared the hierarchical clustering accuracy, ARI and NMI. Both measurements show that with 0.6054 ARI and 0.7892 NMI, I-Impute resulted in the best clustering (ARI:0.1937, NMI:0.45), compared to those based on the imputations by SAVER (ARI:0.5253, NMI:0.7085), scImpute (ARI:0.1937, NMI:0.45), or C-Impute (ARI:0.1664, NMI:0.4317) (Fig. 4a, Additional file 1, Table S5).

We next tested the tools on a mouse embryonic stem (ES) cells dataset. This dataset contains 2717 cells of four cell types (mouse ES cells sample 1, mouse ES cells LIF 2 days, mouse ES cells LIF 4 days and mouse ES cells LIF 7 days). Due to the high running time of scImpute on large cells dataset, we randomly selected 200 cells and no sub-populations and genes were excluded during this process. Due to dropout events, 67.0% of read counts in the raw count matrix are zeros. Figure 4b shows that SAVER and I-Impute achieved overwhelmingly better imputation power than other tools. In the 2D t-SNE embedding space, the results from SAVER and I-Impute both separate the 2 days cells (the yellow dot) from the 4 days cells (the green dots) and the 7 days cells (the blue dots) well. From the Silhouette width, adjusted Rand index, and normalized mutual information, we found that I-Impute (ARI:0.7047, NMI:0.7444, SW:0.2275) produced a tighter and more accurate in-cluster structure than SAVER (ARI:0.692, NMI:0.7329, SW:0.2235)(Additional file 1, Table S6). Hence I-Impute was able to allow identification of the cell sub-populations in spite of the 67.0% missing rate.

Finally, we performed test with a mouse Aortic Leukocyte cells dataset. This dataset contains 378 cells of six cell types (B cells, T cells, T memory cells, Macrophages, Nuocytes, and Neutrophils). Due to dropout events, 91.2% of read counts in the raw count matrix are zeros. Both SAVER and I-Impute grouped the T memory cells (the yellow dots) into big cluster, while in raw data and other imputed matrices, T memory cells are separated into different clusters (see Fig. 4c). In this test, I-Impute gave a silhouette width of 0.0711, which is poorer than the result from SAVER. Nevertheless, I-Impute outperformed all other tools in hierarchical clustering tasks with the highest ARI (0.522) and NMI (0.7728) (Additional file 1, Table S7).

## Discussion

In this paper, we introduced I-Impute, which is designed to impute scRNA missing entries iteratively. Experiments using synthetic and real data demonstrated I-Impute to be particularly suited for cell subpopulation discovery.

There are some advantages of I-Impute compared with scImpute and SAVER. First, I-Impute produces results which will be treated consistently when they are given back as input, and the imputed matrix are of tighter hierarchical structure. Second, scImpute requires the user to decide the cell groups number *K* and assign cells in the same group equal weights during imputation, whereas I-Impute does not require such a hyper-parameter *K* but instead builds a continuous affinity matrix by leveraging on the Gaussian kernel. Last but not least, Lasso regression makes unimportant weights zero, which can help to filter the distant cells for the regression.

Concerning the hyper-parameter pruning, the parameter *t* denotes the threshold of dropout probabilities. We have conducted experiments to guide the pruning. The result in Additional file 1, Fig. S2 suggests that the value of parameter *t* should not be too small, and *t*=0.5 is adequate as the default setting.

## Conclusions

Imputation is an essential step in the use of scRNA-seq. In this work we introduced an imputation criterion called self-consistency and demonstrated the effectiveness of this criterion with an iterative imputation tool called I-Impute. Experiments on simulation data and real data sets showed I-Impute to be highly feasible in imputation and in the discovery of cell sub-population.

## Methods

### C-Impute

I-Impute utilizes a subroutine called C-Impute, which performs imputation with an objective function based on continuous similarity and Lasso penalty (see **Fig.** 1a). The following describes this subroutine.

#### Data prepossessing

The input of C-Impute is a count matrix ${{{\dot {\boldsymbol {X}}}^{C}} \in M \times N_{total}}$ which contains rows as genes and columns as cells, where *M* and *N*_*total*_ represent the total number of genes and cells correspondingly. The dropout values are replaced by zero counts.

First, C-Impute performs normalization, dimension reduction, and outlier removal as in scImpute [18]. This results in a matrix ***X***∈*M*×*N* and ***Z***∈*K*×*N*, where *K* is the reduced dimensionality of metagenes, *N* is the number of remained cells.

#### Affinity matrix constructing

From ***Z***, a cell affinity matrix ***A***∈*N*×*N* is computed with Euclidean distance and Gaussian Kernel:

1 $$\begin{array}{*{20}l} \text{Dist}(i, j) &= \left|\left|{\boldsymbol{Z}}^{\top}_{i} - {\boldsymbol{Z}}^{\top}_{j}\right|\right|^{2}_{F} \end{array} $$

2 $$\begin{array}{*{20}l} \sigma_{i} &= \text{Dist}(i,k) \end{array} $$

3 $$\begin{array}{*{20}l} {\boldsymbol{A}}_{ij} &= \left\{\begin{array}{ll} \exp^{-\frac{\text{Dist}(i, j)}{2\sigma_{i}^{2}}}, \text{ Dist}(i, j) \leq \sigma_{i}, \\ 0, \text{ Dist}(i, j) > \sigma_{i}. \end{array}\right. \end{array} $$

where *i*,*j* represent two different cell indices, ${\boldsymbol {Z}}^{\top }_{i}$ and ${\boldsymbol {Z}}^{\top }_{j}$ indicate the principle components of *i*-th and *j*-th cell respectively, ||·||_*F*_ is the Frobenius norm. For the *i*-th cell, the kernel width will be set to the distance between it and its *n*-nearest neighbor, cell *k*, which stands for the cell whose distance to cell *i* is *n*-th smallest in all other cells, where *n* is a hyper-parameter.

#### Identification of dropout values and calculating dropout rate

With preprocessed gene expression matrix ***X***, we utilize a statistical model to infer which entries are influenced by the dropout effects. Instead of treating all zero values as missing entries, we use the Gamma-Normal mixture model to learn whether a zero observation originates from dropout or not. We use the Normal distribution to present the actual gene expression level and Gamma distribution to take the dropout events into account. Since the preprocessed matrix ***X*** is no longer of integral values, we cannot adopt zero-inflated negative binomial (ZINB) distribution.

For the *i*-th gene and its observed value *x* in prepossessed gene profiling ***X***_*i*_, the Gamma-Normal mixture model will be:

4 $$\begin{array}{*{20}l} {}\begin{aligned} &f_{\text{Gamma-Normal}}\left(x; {\boldsymbol{\pi}}_{i}, {\boldsymbol{\alpha}}_{i}, {\boldsymbol{\beta}}_{i}, {\boldsymbol{\mu}}_{i}, {\boldsymbol{\sigma}}_{i}\right) \\ =&{\boldsymbol{\pi}}_{i} \text{Gamma}\left(x;{\boldsymbol{\alpha}}_{i},{\boldsymbol{\beta}}_{i}\right)+\left(1-{\boldsymbol{\pi}}_{i}\right)\text{Normal}\left(x;{\boldsymbol{\mu}}_{i}, {\boldsymbol{\sigma}}_{i}\right) \end{aligned} \end{array} $$

where ***π***_*i*_ is the dropout rate of gene *i*, ***α***_*i*_ and ***β***_*i*_ is the shape and rate parameter of Gamma distribution respectively, ***μ***_*i*_ and ***σ***_*i*_ are the mean and standard deviation of Normal distribution. The estimated model parameters $\hat {{\boldsymbol {\pi }}}, \hat {{\boldsymbol {\alpha }}}, \hat {{\boldsymbol {\beta }}}, \hat {{\boldsymbol {\mu }}}$, and $\hat {{\boldsymbol {\sigma }}}$ are obtained by Expectation-Maximization (EM) algorithm. Then, we can calculate the dropout probability matrix ***D***∈*M*×*N*.

5 $$\begin{array}{*{20}l} {\boldsymbol{D}}_{ij}= \frac{{\boldsymbol{\pi}}_{i} \text{Gamma}\left({\boldsymbol{X}}_{ij}; {\boldsymbol{\alpha}}_{i}, {\boldsymbol{\beta}}_{i}\right)}{f_{\text{Gamma-Normal}}\left({\boldsymbol{X}}_{ij}; {\boldsymbol{\pi}}_{i}, {\boldsymbol{\alpha}}_{i}, {\boldsymbol{\beta}}_{i}, {\boldsymbol{\mu}_{i}}, {\boldsymbol{\sigma}}_{i}\right) } \end{array} $$

This mixture model enables the identification of whether an observed value is a dropout value or not, since a zero value can be either caused by a technical error or may reflect the actual expression value. If a gene has high expression and low variation in most of its similar cells, a zero count will have high dropout probability and more likely to be a dropout value; otherwise, the zero value may exhibit real biological variability [18].

#### Imputation of dropout values

To impute the gene expression levels, we first define a hyper-parameter *t* which is used as the threshold to determine if ***X***_*ij*_ is a dropout event. An entry of dropout probability less than *t* is considered a real observation, in which case its value is retained. Otherwise, while values with dropout probability higher than *t* will be replaced by imputation result. We perform imputation by linear regression weighted by dropout probability and cell affinity.

6 $$\begin{array}{*{20}l} \hat{{\boldsymbol{X}}}_{ij} &= \left\{\begin{array}{lc} {\boldsymbol{X}}_{ij}, {\boldsymbol{D}}_{ij} < t, \\ \left(\left(1-{\boldsymbol{D}}_{\bar{j}}^{\top}\right)\circ\left({\boldsymbol{A}}_{j\bar{j}} \odot {\boldsymbol{X}}^{\top}_{\overline{j}}\right)\right){\boldsymbol{B}}^{\top}_{j}, {\boldsymbol{D}}_{ij} \geq t. \end{array}\right. \end{array} $$

where ${\boldsymbol {D}}^{\top }_{j}$ and ${\boldsymbol {X}}^{\top }_{j}$ are the *j*-th column of ***D*** and ***X*** respectively. The ∘ operator is the Hadamard product which follows (***P***∘***Q***)_*ij*_=***P***_*ij*_***Q***_*ij*_. $\bar {j}$ denotes all indices except index *j*, thus ${\boldsymbol {D}}^{\top }_{\bar {j}}$ and ${\boldsymbol {X}}^{\top }_{\bar {j}}$ denotes the sub-matrix of ***D*** and ***X*** which contains all cells except the *j*-th cell, respectively. ${\boldsymbol {A}}_{j\bar {j}}$ stores the pairwise affinity between *j*-th cell and all other cells; ${\boldsymbol {X}}^{\top }_{\bar {j}}$ is a sub-matrix of ***X*** which contains all cells except the *j*-th cell. ⊙ operator represents the vector and matrix multiplication, e.g. (***p***⊙***Q***)_*ij*_=***p***_*i*_***Q***_*ij*_. Leveraging $\left (1-{\boldsymbol {D}}^{\top }_{j}\right)\circ {\boldsymbol {X}}^{\top }_{j}$ as target indicates that genes with high dropout probability in *j*-th cell will not contribute to optimization. Furthermore, the multiplication of $\left (1-{\boldsymbol {D}}_{\bar {j}}^{\top }\right)$ and ${\boldsymbol {A}}_{j\bar {j}}$ ensures that the information is only borrowed from the trusted genes with low dropout probabilities in the similar cells. Non-negative weights ${\boldsymbol {B}}^{\top }_{j}$ are extra contributions of all other cells learned from regression.

For *j*-th cell, the objective is:

7 $$\begin{array}{*{20}l} \begin{aligned} \underset{{\boldsymbol{B}}^{\top}_{j}}{\min} &\sum\limits_{j = 1}^{N}||\left(1-{\boldsymbol{D}}^{\top}_{j}\right)\circ{\boldsymbol{X}}^{\top}_{j} \\ &- \left[\left(1-{\boldsymbol{D}}_{\bar{j}}^{\top}\right)\circ\left({\boldsymbol{A}}_{j\bar{j}} \odot {\boldsymbol{X}}^{\top}_{\overline{j}}\right)\right]{\boldsymbol{B}}^{\top}_{j}||_{F}^{2} \\ &+ \lambda \left|\left|{\boldsymbol{B}}^{\top}_{j}\right|\right|_{1},\\ \text{ sub}&\text{ject to }{\boldsymbol{B}}^{\top}_{j} \geq 0 \\ \end{aligned} \end{array} $$

$\mathcal {L}$1 is applied to avoid over-fitting and further ensure that the imputation borrow information from the cell’s most similar neighbors.

Assume $y \in \mathbb {R}^{M} = \left (1-{\boldsymbol {D}}^{\top }_{j}\right)\circ {\boldsymbol {X}}^{\top }_{j},\beta \in \mathbb {R}^{N} = {\boldsymbol {B}}_{j}^{\top }, X \in \mathbb {R}^{M \times N} = \left (1-{\boldsymbol {D}}_{\bar {j}}^{\top }\right)\circ \left ({\boldsymbol {A}}_{j\bar {j}} \odot {\boldsymbol {X}}^{\top }_{\overline {j}}\right)$, for each *j*-th cell we can simplify the objective to non-negative least squares lasso regression $\min _{\beta }\left |\left |y - X\beta \right |\right |_{2}^{2} + \lambda \left |\left |\beta \right |\right |_{1},\beta \geq 0$, and solve it by coordinate descent [31].

### I-Impute

As mentioned, I-Impute performs a self-consistent imputation on scRNA-seq data. The method is as illustrated in **Fig.** 1b. I-Impute utilizes C-Impute to iteratively refine SAVER processed data. After a few iterations, the result converges to a self-consistent matrix (<*θ*) and is given as I-Impute’s output.

We define self-consistency of a functional mapping *f*:*x*→*x* given by input data ***X***∈*M*×*N*:

8 $$\begin{array}{*{20}l} \begin{aligned} {\boldsymbol{X}}_{output} &= f({\boldsymbol{X}}) \\ \text{self-consistency}\,(f; {\boldsymbol{X}}) &= \frac{\left|\left|{\boldsymbol{X}}_{output} - f\left({\boldsymbol{X}}_{output}\right)\right|\right|^{2}_{F}}{M \times N} \\ \text{self-consistency}\,(f; {\boldsymbol{X}}) &< \theta \rightarrow f \text{ is self-consistent} \end{aligned} \end{array} $$

### Evaluation metrics

#### Adjusted rand index and normalized mutual information

The adjusted Rand index (ARI) [32] and normalized mutual information (NMI) [33] are adopted as clustering accuracy. They measure the similarity between a clustering result and the actual clusters. A value close to 0 indicates random labeling or no mutual information, and a value of 1 demonstrates 100% consistency between the clustering and the actual clusters.

#### Silhouette width

The silhouette width (SW) measures the similarity of a sample to its class compared to other categories [34]. It ranges from -1 to 1. A higher silhouette value suggests a more appropriate clustering. A silhouette value near 0 indicates overlapping clusters and a negative value indicates that the clustering has been performed incorrectly. We adopted the silhouette width to evaluate the model’s imputation power. We used the ground-truth subtype classes as the input cluster labels.

### Simulation and benchmark settings

Splatter are used to generate simulated scRNA-seq data. The parameters used for our simulation dataset are nGroups=3, nGenes=2000, batchCells=150, seeds=42, dropout.type=“experiment”, dropout.shape=-1 and dropout.mid=2, 3, 5 for three different dropout rate data.

SAVER and scImpute are the state-of-the-art tools which I-Impute is compared against. For the SAVER R package, we used the “saver” function with the parameters ncores=12 and estimates.only=TRUE to perform the imputation tasks. The parameters for scImpute are drop_thre=0.5, ncores=10, Kclusters=(number of true clusters in input data).

On synthetic data, I-Impute configuration is n=40, normalize=False, and iteration=True. On real data sets, I-Impute configuration is n=40, and iteration=True when tested with the mouse Bladder cell dataset and ES cell dataset, and is n=20, and iteration=True when tested with the mouse Aortic Leukocyte cell dataset.

## Supplementary information

**Additional file 1** The PDF file includes all the supporting materials for the manuscript.

## Data Availability

The real scRNA-seq datasets analysed during the current study are all publicly available. The mouse ES cell dataset [35] was downloaded from the Gene Expression Omnibus (GEO) with the accession code GSE65525. The mouse Bladder cell dataset and Aortic Leukocyte cell dataset were downloaded from the PanglaoDB [36] with the accession code SRS3044239and SRS2747908respectively. The Python package I-Impute is freely available at https://github.com/xikanfeng2/I-Impute.

## Abbreviations

scRNA-seq: Single-cell RNA-sequencing
ARI: Adjusted Rand Index
NMI: Normalized Mutual Information
SW: Silhouette Width
RMSE: Root Mean Square Error
