# Supplementary material for: I-Impute: a self-consistent method to impute single cell RNA sequencing data
Source: BMC Genomics. 2020 Nov 18;21(Suppl 10):618. doi: 10.1186/s12864-020-07007-w (PMC7677776; doi:10.1186/s12864-020-07007-w)
Supplement: Supplementary file 1 — Additional file 1 The PDF file includes all the supporting materials for the manuscript. [file 12864_2020_7007_MOESM1_ESM.pdf]

I-Impute: a self-consistent method to impute single cell RNA sequencing data
Additional file 1

Xikang Feng+, Lingxi Chen+, Zishuang Wang, Shuai Cheng Li*

November 18, 2019

Contents

1 Supplementary Figures **2**

2 Supplementary Tables **3**

1 Supplementary Figures

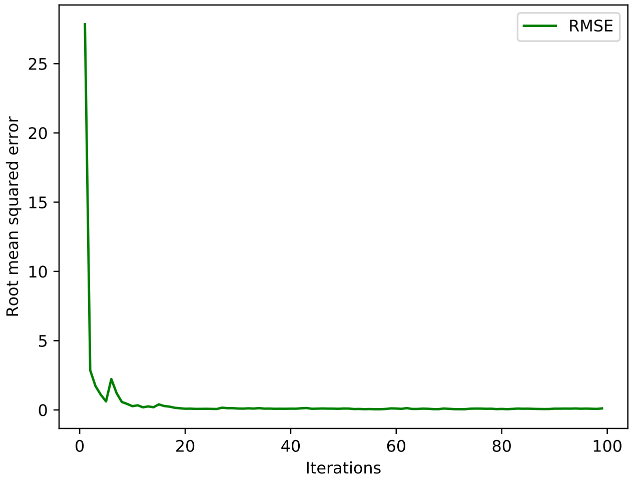

Figure S1: Iteration analysis based on synthetic data (88.45% dropout). The root mean squared error (RMSE) is stable below 0.1.

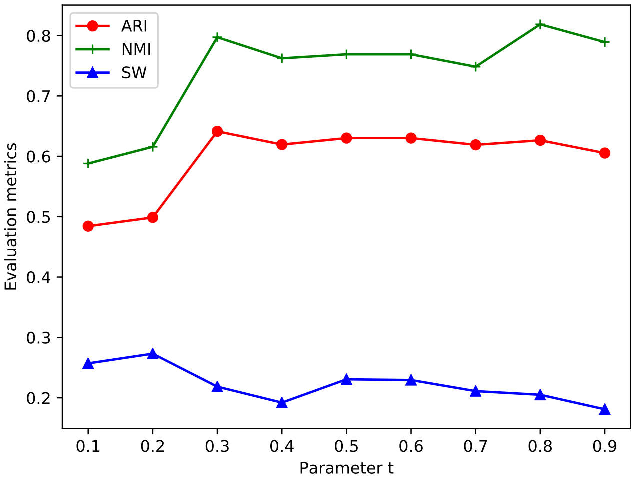

Figure S2: Clustering results of imputed data based on mouse Bladder cells dataset when different values of parameter t are used in I-Impute. This result suggests that the value of parameter t should not be too small. The default value of parameter t is 0.5.

2 Supplementary Tables

Table S1: Imputation performance on synthetic data (88.45% dropout)

Method	ARI	NMI	SW
Raw	0.5161	0.5787	0.2627
88.45% Dropout	0.4363	0.5467	0.0862
SAVER	0.7473	0.7145	0.1075
scImpute	0.0531	0.0989	0.0742
C-Impute	0.6860	0.7512	0.1705
I-Impute	**0.8721**	**0.8521**	**0.2429**

Table S2: Imputation performance on synthetic data (63.29% dropout)

Method	ARI	NMI	SW
Raw	0.5161	0.5787	0.2627
63.29% Dropout	0.3105	0.4115	0.1910
SAVER	0.9784	0.9700	0.2739
scImpute	0.2033	0.3904	0.2054
C-Impute	0.9644	0.9509	0.2496
I-Impute	**1.0**	**1.0**	**0.3908**

Table S3: Imputation performance on synthetic data (45.16% dropout)

Method	ARI	NMI	SW
Raw	0.5161	0.5787	0.2627
45.16% Dropout	0.6000	0.6306	0.2137
SAVER	0.9801	0.9710	0.3292
scImpute	0.3596	0.4962	0.2365
C-Impute	0.4727	0.5551	0.2566
I-Impute	**0.9801**	**0.9710**	**0.4123**

Table S4: Pearson correlation result on synthetic data

Method	88.45% Dropout	63.29% Dropout	45.16% Dropout
SAVER	0.5836	0.7915	0.8764
scImpute	0.6451	0.8582	0.9263
C-Impute	0.7611	0.8873	0.9305
I-Impute	**0.7812**	**0.8993**	**0.9357**

Table S5: Imputation performance on mouse Bladder cells data

Method	ARI	NMI	SW
Raw	0.1937	0.4500	0.0737
SAVER	0.5253	0.7085	0.0621
scImpute	0.1937	0.4500	0.0686
C-Impute	0.1664	0.4317	0.0741
I-Impute	**0.6054**	**0.7892**	**0.1758**

Table S6: Imputation performance on mouse ES cells data

Method	ARI	NMI	SW
Raw	0.2410	0.5160	0.0353
SAVER	0.6920	0.7329	0.2235
scImpute	0.3574	0.5258	0.0418
C-Impute	0.2410	0.5160	0.0411
I-Impute	**0.7047**	**0.7444**	**0.2275**

Table S7: Imputation performance on mouse Aortic Leukocyte cells data

Method	ARI	NMI	SW
Raw	0.3463	0.6352	0.0290
SAVER	0.3605	0.6837	**0.1075**
scImpute	0.3377	0.6237	0.0358
C-Impute	0.3427	0.6398	0.0206
I-Impute	**0.5220**	**0.7728**	0.0711
